# Psychometric assessment of the Post- Secondary Student Stressors Index (PSSI)

**DOI:** 10.1186/s12889-019-7472-z

**Published:** 2019-08-19

**Authors:** Brooke Linden, Heather Stuart

**Affiliations:** 1 Department of Public Health Sciences, Abramsky Hall, Queen’s University, 21 Arch Street, Kingston, ON Canada; 2 Department of Public Health Sciences, Bell Canada Mental Health and Anti-Stigma Research Chair, Abramsky Hall, Queen’s University, 21 Arch Street, Kingston, ON Canada

**Keywords:** Mental health, Stress, Post-secondary, Scale development, Psychometrics, Health promotion

## Abstract

**Background:**

Previous research has linked excessive stress among post-secondary students to poor academic performance and poor mental health. Despite attempts to ameliorate mental health challenges at post-secondary institutions, there exists a gap in the evaluation of the specific sources of stress for students within the post-secondary setting.

**Methods:**

The goal of this study was to develop a new instrument to better assess the sources of post-secondary student stress. Over the course of two years, the Post-Secondary Student Stressors Index (PSSI) was created in collaboration with post-secondary students as co-developers and subject matter experts. In this study, we used a combination of individual cognitive interviews (*n* = 11), an online consensus survey modeled after a traditional Delphi method (*n* = 65), and an online pre- (*n* = 535) and post-test (*n* = 350) survey to psychometrically evaluate the PSSI using samples of students from Ontario, Canada. We collected four types of evidence for validity, including: content evidence, response processes evidence, internal structure evidence, and relations to other variables. The test-retest reliability of the instrument was also evaluated.

**Results:**

The PSSI demonstrated strong psychometric properties. Content validation and response processes evidence was derived from active student involvement throughout the development and refinement of the tool. Exploratory factor analysis suggested that the structure of the PSSI reflects the internal structure of an index, rather than a scale, as expected. Test-retest reliability of the instrument was comparable to existing, established instruments. Finally, the PSSI demonstrated good relationships with like measures of stress, distress, and resilience, in the hypothesized directions.

**Conclusions:**

The PSSI is a 46-item inventory that will allow post-secondary institutions to pinpoint the most severe and frequently occurring stressors on their campus. This knowledge will facilitate appropriate targeting of priority areas, and help institutions to better align their mental health promotion and mental illness prevention programming with the needs of their campus.

## Background

Over the past decade, chronic stress and mental health problems among Canadian post-secondary students have become a main focus of attention. Research has linked excessive stress among post-secondary students to a number of negative outcomes, including deteriorated mental health [1–3] and poor academic performance [4]. The most recent Canadian edition of the National College Health Assessment II revealed a substantial prevalence of both stress and common mental illnesses, such as anxiety and depression (formally diagnosed, or self-reported through the use of screening tools) [4]. Prevalence estimates for self-reported symptoms of anxiety and depression increased between the 2013 and 2016 iterations of the survey [4].

While many post-secondary institutions have attempted to ameliorate these issues by increasing on-campus treatment options, few have managed to develop effective upstream services, such as mental health promotion and mental illness prevention [5, 6]. In fact, in a recent survey completed by representatives of post-secondary institutions across Canada, only 70% believed that students were well-informed about mental health issues and available services on campus, while almost all representatives indicated they thought their campus could benefit from expanding mental health promotion and outreach activities [6, 7]. Existing mental health promotion and mental illness prevention may be improved with better targeting of the main issues faced by students, but the ability to do so hinges on an improved understanding of student-specific stress. Improving upstream approaches targeting mental health promotion and stress reduction may help to alleviate the burden of mental health problems among student populations, as well as the demand currently placed on overtaxed campus treatment services.

Existing instruments designed to evaluate post-secondary student stress can be improved. Few have involved a diverse sample of students in the development process (e.g., engaging only students in a particular year, level, or program of study), while others have been developed too narrowly (e.g., items developed based solely on literature, little consideration for student input) or too broadly (e.g., including stress-related items not relevant to, or modifiable by the post-secondary institution). This research details the development and preliminary validation of the Post-Secondary Student Stressors Index (PSSI), an index of student-specific stressors developed and comprehensively validated using a ‘for-students-by-students’ approach.

## Methods

The PSSI was developed and validated with students, for students through a series of steps. The initial pool of items was developed by students through the use of an online survey and focus group interviews in 2018, the results of which are detailed elsewhere [8]. For each stressor on the instrument, respondents were asked to indicate the severity of stress experienced, and the frequency with which they experienced it. Response options ranged from a scale of 1 (‘not stressful’ and ‘rarely’) to 4 (‘very stressful’ and ‘almost always’), with higher ratings indicating a greater level of stress resulting from an item. An additional option to indicate ‘N/A’ was also available in the event that a stressor didn’t happen or was otherwise not applicable.

In this study, we used a number of methods to refine the preliminary index and collect evidence assessing validity and reliability. Validity is described as a process by which we determine the degree of confidence we can place on the inferences we make about people based on their scores on an instrument [9]. Reliability refers to the consistency of test scores within a particular population. According to the *Standards for Educational and Psychological Testing* (“the *Standards*”), validation of an instrument requires the accumulation of evidence from five sources: content; response processes; internal structure; relations to other variables; and test consequences [10]. This article reports the collection of the first four types of validity evidence for the PSSI. Analyses were completed using R, Version 3.4.1. This research received ethics clearance from Queen’s University’s Health Sciences and Affiliated Teaching Hospitals Research Ethics Board.

### Response processes evidence

Response process validation evidence provides empirical evidence of the extent to which participants’ responses to the items on an instrument align with the construct under study [10]. We collected response processes evidence from 11 individual cognitive interviews conducted by the first author using a think-aloud technique with verbal probing [11]. Participants were asked to complete the preliminary version of the PSSI on the interviewer’s desktop computer, explaining their interpretation of questions and the reasoning behind their response selections. Note that the interviewer must be physically present during cognitive interviews in order to pick up on non-verbal communications, including body language and facial expressions. Notes were taken electronically during the interviews, and immediately reviewed afterward. Problems identified by participants were recorded using problem codes developed by Willis [12], described in Table 1. Following each interview, corrective action was taken. This “waterfall” method of correction ensured that every interview provided maximum value to the improvement of the instrument. The only exception was with items recommended for removal; these were carefully considered after all interviews had been completed.

**Table 1:** Cognitive Interviewing Problem Codes

Problem Code	Description	Corrective Action
[1] Clarity	Respondent was confused by the item wording, or felt that it was ambiguous.	Change in wording
[2] Relevance	Respondent felt that the item addressed an issue not relevant to student stress.	Change in wording Delete the item
[3] Recall	Respondent had trouble recalling or remembering information required for answer.	Change in wording Change response categories
[4] Redundancy	Respondent felt the item did not add value in the context of other items.	Delete the item
[5] Bias	Respondent felt the wording of the item encouraged them to respond in a certain way.	Change in wording Change order of items
[6] Comprehensive	Respondent felt there was an indicator missing from the list.	Add an item

### Content evidence

Content evidence refers to the degree to which the items on an instrument represent the area of interest [10]. In this study, content evidence was collected through the use of an online consensus survey modeled after a traditional Delphi method [13]. In the context of this study, we considered our subject matter “experts” to be post-secondary students, given their lived experience with stress in the post-secondary setting. Participants were provided with the PSSI and asked to rate the relevance and clarity of each item on two adjectival scales, anchored by 1 (not at all relevant/clear) and 4 (very relevant/clear) [14]. Finally, participants were also invited to add any stressors they felt were missing from the PSSI. We used responses to compute the content validity index for each item (I-CVI), calculated by dividing the number of respondents who rated each item as a 3 (somewhat relevant/clear) or 4 (very relevant/clear) by the total number of respondents [15]. The I-CVI expresses the proportion of agreement on the relevancy and clarity of each item, and lies between 0 and 1 [16]. Based on recommendations in the literature, items with relevance I-CVIs of 0.7 or greater were retained [15, 17]. Clarity ratings were used to assess whether further revision of items was required for readability and comprehension. Content validity indices for the scales in their entirety (S-CVIs) were calculated by taking the average of the relevance I-CVIs for retained items only.

### Internal structure evidence and relations to other variables

A pilot test of the PSSI was conducted among a random sample of over 500 students at an Ontario university in the winter of 2019 to facilitate the collection of internal structure evidence and examine test scores’ relations to other variables. An online survey was developed, comprising the PSSI, eight demographic questions, and three validated scales evaluating like constructs. The 10-item Perceived Stress Scale (PSS-10) is a brief scale designed to measure general, or “global” stress [18]. The Kessler Psychological Distress Scale (K10) is a 10-item scale designed to measure general distress [19]. Both the PSS-10 and K10 have demonstrated consistent psychometric properties in a number of samples and settings and have previously been used among post-secondary populations. The 10-item Connor-Davidson Resiliency Scale (CD-RISC) is a scale designed to measure resilience, conceptualized by the authors as a measure of “stress coping ability” [20, 21]. This 10-item version of the scale was created using samples of undergraduate students, and has shown strong psychometric properties [21]. A second survey was sent to participants who completed the first survey approximately two weeks later containing only the PSSI and the PSS-10. Responses on each survey were matched using anonymous, unique identifiers in order to evaluate the test-retest reliability of test scores.

Responses to the first online survey were used to assess the internal structure of the PSSI. Internal structure evidence refers to the degree to which the relationships among items in the instrument are consistent with what is expected of the construct under study [10]. The PSSI was designed to be an index, rather than a scale so individual stress items were conceptualized as causal indicators (e.g., “causes” of stress), rather than effect indicators (e.g., “effects” of stress), as would be the case with a scale [14, 22]. As there is no assumption about the homogeneity of items within an index, we used an exploratory factor analysis to determine whether the PSSI had the dimensionality of an index, as hypothesized. In other words, we hypothesized that a factor analysis would reveal no clear “groupings” of stressors loading on distinct factors. Additionally, we expected some items to be correlated, and others not, as is indicative of an index [22]. To assess the test-retest reliability of the instrument, we used the matched responses on the first and second surveys, examining correlations between scores on the PSSI across the two-week interval. We hypothesized that students’ stress levels would remain fairly static over this two-week period, and would consider a correlation coefficient of 0.7 to indicate good test-retest reliability [9].

Relations to other variables evidence refers to whether the test scores from the instrument correlate significantly, and in the direction expected, with like and unlike constructs measured by existing, valid instruments [10]. We assessed the PSSI test scores’ relationships to other variables, investigating correlations between the scores on the PSS-10, K10, and CD-RISC. As our goal with the PSSI was to develop an instrument to evaluate student stress specific to the post-secondary environment, we hypothesized that the PSSI would have a positive, moderate correlation with the PSS-10. A very strong correlation with a global stress measure would indicate that the PSSI had been measured too generally. Similarly, we hypothesized that the PSSI would have a positive, moderate correlation with the K-10, a global measure of distress, as the constructs of stress and distress are closely related [23]. Finally, we expected that participants with higher scores on the PSSI would score lower on the CD-RISC (e.g., be less resilient), resulting in a negative correlation.

## Results

### Response processes evidence

Eleven cognitive interviews were conducted over a two-month period. Participants were almost evenly split between the undergraduate (45%) and graduate (55%) levels of study, were mostly female (63.6%), and studied in a variety of different areas. Problem codes were used to identify issues with the instrument. Table 2 shows the number of problem codes marked in each category for each participant.

**Table 2:** Number of Problems Identified by Cognitive Interview Participants

Interviewee	Clarity	Relevance	Recall	Redundancy	Bias	Comprehensive	Response Categories
1 (PHD1)	5			1		1	1
2 (UG3)	4			1		2	
3 (UG3)	2						1
4 (MA1)	2						1
5 (UG3)	2	1					
6 (UG2)	2						
7 (PHD2)	1	1					1
8 (MA2)	2	1					
9 (MA2)	2						
10 (UG4)	1						
11 (PHD2)	0						

Note**.** *UG* undergraduate, *MA* Master’s level graduate student, *PHD* PhD level graduate student. Number indicates year of study

Examples of interview responses are included in Table 3. No problems with *recall* or *bias* were identified by any of the participants. The majority of the problems identified were regarding *clarity*, with participants asking for clarification on items, and suggesting rewordings to make items more explicit. For example, one participant explained that the item “completing assignments” should be clarified to emphasize having to manage multiple assignments simultaneously. As a result, this item was changed to “having multiple assignments due around the same time.” Both *redundancies* were identified among stressors within the learning environment theme. Students suggested that “asking my professor a question,” “asking my professor to remark something,” and “asking my professor for clarification on a grade” were largely addressing the same stressor: interaction with faculty. Therefore, these items were collapsed into a single item. Three items were added as per participants’ recommendations to ensure *comprehensiveness* of the index. Students questioned the *relevance* of some items within the personal stressors theme to one’s experience as a student (e.g., “worrying about my personal appearance”), though as these problems were rarely identified, the items were ultimately left in for the subsequent phase of testing. Finally, several students struggled with the response options used to evaluate frequency. While these options were initially more specific (e.g., a few times per year, per month, per week), they were altered to be more general (e.g., rarely, sometimes, regularly) in response to participants’ feedback. Additionally, the highest frequency response option was changed from “always” to “almost always” to dissuade participants from shying away from the extreme option.

**Table 3:** Examples of Cognitive Interview Responses

Item	Discussion
Completing assignments	*Interviewer*: I noticed you paused … what were you thinking when you read that item?
	*Respondent*: Well […] “completing assignments” is stressful in the sense that you have to do it, but if the assignment is very clear, then it’s not as stressful. Having a bunch of assignments all piling up [at the same time] … now that is stressful.
Lack of guidance from professor	*Interviewer*: Can you tell me what you were thinking about when you responded to that item?
	*Respondent*: If I ask for clarification on something and they say “just do what’s in the syllabus” then it’s like… okay, why do I even bother?
Waiting to receive grades/marks	*Interviewer*: I see you went back and changed your answer on that item … can you tell me why?
	*Respondent*: At first I thought, yeah… pretty stressful, [but it’s] only stressful if the next assignment is due soon and I don’t have the [other] grade back yet … I need to know where I’m at, you know?
Pressure to succeed	*Interview: [Participant scoffed while answering ‘extremely stressful’]* What were you thinking when you responded to that item?
	*Respondent*: What if I can’t get a job after my PhD? Was it all a waste of time? A lot of my friends, my [significant other], think that I’m going to be successful just because I’m doing my PhD… but what if I fail? I’m letting so many people down. I think about that every day.
Feeling like my peers are smarter than I am	*Interviewer:* What made you answer that way?
	*Respondent*: […] when you hear someone contribute to conversation and you’re like… ‘Oh my God, how did they think of that? I don’t belong here.’

### Content evidence

Using a snowball sampling approach, we were successful in recruiting a sample of 65 post-secondary students to serve as our panel of participants (Table 4). Our goal at this stage was to recruit a demographically varied sample of students across provinces and areas of study in order to gain a broader perspective on student stress. The majority of participants were female (76.6%) university students (95.7%) from Ontario (79.5%), at the undergraduate (30.4%) or master’s degree level (34.8%), with an average age of 24 years (SD = 3.5).

**Table 4:** Demographic Characteristics of Consensus Survey Sample (*n* = 47)

Variable	Number n	Percent %
Province		
Ontario	35	79.5
Quebec	6	13.6
Nova Scotia	1	2.3
Alberta	1	2.3
British Columbia	1	2.3
Type of Post-Secondary Institution		
University	45	95.7
College	2	4.3
Sex		
Female	36	76.6
Male	11	23.4
Age Group		
19–21 years	11	23.4
22–25 years	20	42.6
26–29 years	15	32.0
30+ years	1	2.0
Level of Study		
1st – 2nd year Undergraduate	5	10.8
3rd – 4th year Undergraduate	9	19.6
Masters Student	16	34.8
Doctoral Student	6	13.0
Professional Program	10	21.7
Program of Study		
Health Science	12	25.5
Social Science	8	17.0
Medicine	6	12.7
Nursing	4	8.5
Education	3	6.4
Speech-Language Pathology	3	6.4
Business	3	6.4
Engineering	2	4.3
Science	2	4.3
Other	4	8.5

Note. Valid percents reported. Missing data on demographic variables (*n* = 18)

A total of 38 items demonstrated I-CVIs < 0.7, the recommended cut off for retention [15, 17]. All items met the cutoff for clarity. While some items’ relevancy ratings did not meet the I-CVI cutoff, we carefully considered these, choosing to retain those that we considered were important to the overall comprehensiveness of the instrument. For example, some items were retained due to being prevalent in focus groups held with students during the item pool development phase of this program of research (e.g., worrying about getting into a new program after graduation, feeling pressured to socialize). Others were retained if we thought the item CVI might have been higher had the sample we used contained more students to whom the item applied (e.g., having to take student loans, and working on one’s thesis). Items addressing sexual harassment and instances of discrimination on campus were also retained despite falling below the threshold, as we considered these were important potential student experiences of campus culture that every institution should seek to monitor. Table 5 displays the retained items along with their relevance and clarity content validity indices, organized by domain of stress.

**Table 5:** Content Validity Indices for PSSI Items, by Theme

Item	Relevance I-CVI	Clarity I-CVI
Academic		
a) Preparing for exams	0.99	0.95
b) Writing exams	0.92	0.97
c) Writing multiple exams on the same day	0.89	0.98
d) Final exams worth 50% or more	0.86	0.95
e) Heavily weighted assignments	0.79	0.89
f) Having multiple assignments due around the same time	0.94	0.98
g) Managing my academic workload	0.82	0.83
h) Maintaining a high GPA	0.91	0.94
i) Working on my thesis	0.69*	0.84
j) Performing well at my professional placement (i.e., practicum)	0.78	0.88
Learning Environment		
a) Poor communication from professor	0.82	0.80
b) Unclear expectations from professor	0.89	0.93
c) Lack of guidance from professor	0.80	0.83
d) Meeting my thesis/placement supervisor’s expectations	0.80	0.98
e) Lack of mentoring from my thesis/placement supervisor	0.84	0.94
Campus Culture		
a) Adjusting to the post-secondary lifestyle	0.75	0.88
b) Adjusting to my program	0.69*	0.92
c) Academic competition among my peers	0.71	1.00
d) Feeling like I’m not working hard enough	0.87	0.96
e) Feeling like my peers are smarter than I am	0.87	1.00
f) Pressure to succeed	0.98	0.98
g) Sexual harassment on campus	0.62*	0.94
h) Discrimination on campus	0.64*	0.94
Interpersonal		
a) Making new friends	0.80	0.98
b) Maintaining friendships	0.80	0.96
c) Networking with the ‘right’ people	0.76	0.92
d) Feeling pressured to socialize	0.66*	0.94
e) Balancing social life with academics	0.88	0.98
f) Comparing myself to others	0.86	0.98
g) Comparing my life to others on social media	0.76	0.96
h) Meeting other peoples’ expectations of me	0.86	1.00
i) Meeting my own expectations	0.98	0.98
Personal		
a) Getting enough sleep	0.87	0.96
b) Getting enough exercise	0.83	0.98
c) Making sure that I eat healthy	0.79	0.98
d) Having to prepare meals for myself	0.70	1.00
e) Balancing working at my job with academics	0.79	0.98
f) Balancing my extracurriculars with academics	0.83	0.94
g) Feeling guilty about taking time for my hobbies/interests	0.81	0.98
h) Having to take student loans	0.68*	1.00
i) Worrying about paying off debt	0.72	0.98
j) Worrying about getting a job after graduation	0.83	1.00
k) Worrying about getting into a new program after graduation	0.68*	0.98

Note. * Indicates item was retained despite I-CVI < 0.7

### Relations to other variables

A total of 535 participants completed the initial pilot test survey, representing a response rate of 11%. Most participants were female (74.0%), single (64.9%), lived off campus with roommates (62.1%), self-reported their GPA to be between 80 and 89% (41.7%), and studied full-time (92.1%), and at the undergraduate level (65.5%). The majority of participants were between the ages of 19 and 21 years (63.7%), with an overall average age of 24.5 years (*SD* = 7.0). International students made up about 9% of the sample. Of the 535 students, a total of 350 completed the second survey (response rate 65%) with a similar demographic breakdown (Table 6).

**Table 6:** Demographic Characteristics of Pilot Test Sample

Variable	Survey 1 (n = 535)		Survey 2 (n = 350)	
	Number n	Percent %	Number n	Percent %
Sex				
Female	396	74.0	270	77.1
Male	132	24.7	76	21.7
Non-Binary	3	0.6	2	0.6
Prefer not to answer	4	0.7	2	0.6
Age Group				
18–21 years	264	49.3	177	50.6
22–25 years	130	24.3	83	23.7
26–29 years	65	12.1	44	12.6
30+ years	76	14.2	46	13.1
Relationship Status				
Single	347	64.9	234	66.9
In a Relationship	111	20.7	66	18.8
Married or Common-law	68	12.7	42	12.0
Separated, Divorced, or Widowed	2	0.3	2	0.6
Prefer not to answer	7	1.3	6	1.7
Living Arrangement				
Campus residence hall	35	6.4	22	6.3
Other on-campus housing	13	2.4	6	1.7
Off campus with roommates	332	62.1	224	64.0
Off campus alone	69	12.9	48	13.7
Off campus with family	83	15.5	47	13.4
Prefer not to answer	3	0.6	3	0.9
Level of Study				
1st - 2nd year Undergraduate	187	34.9	112	32.0
3rd - 5th year Undergraduate	154	28.8	109	31.1
Graduate (Masters)	72	13.5	47	13.4
Graduate (Doctoral)	53	9.9	37	10.6
Professional Program	47	8.8	30	8.6
Other	21	3.9	14	4.0
Prefer not to answer	1	0.2	1	0.2
Student Status				
Full-time	493	92.1	324	92.6
Part-time	32	6.0	21	6.0
Other^a^	8	1.5	4	1.1
Prefer not to answer	2	0.4	1	0.3
International Student				
No	486	90.8	324	92.6
Yes	48	9.0	25	7.1
Prefer not to answer	1	0.2	1	0.3
Approximate GPA				
90–100%	103	19.3	69	19.7
80–89%	223	41.7	145	41.4
70–79%	147	27.5	98	28.0
60–69%	32	6.0	`17	4.9
0–59%	4	0.7	2	0.6
Prefer not to answer	26	4.9	19	5.4

Note. Valid percents reported

^a^ “Other” category includes non-degree seekers, online students, medical residents

**Fig. 1:**
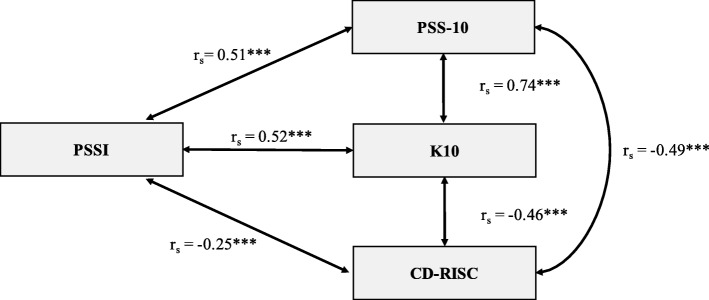
Graphical Depiction of the Correlational Relationships between Instruments

To calculate “scores” on the PSSI, we dichotomized responses to reflect whether participants experienced some level of stress response to stressor (coded as 1) or did not experience or did not find the experience stressful (coded as 0). For each respondent, we then summed the number of stressors experienced to derive an absolute count, which was treated as a “score”. We used the non-parametric Spearman’s rho to calculate correlations between the PSSI scores and those on other instruments, as the data were not normally distributed. Table 7 and Fig. 1 show the correlations between each of the instruments tested. As hypothesized, the PSSI demonstrated a positive, moderate correlation with both the PSS-10 and K-10. As expected, the PSSI also demonstrated a negative correlation with the CD-RISC, indicating that as the number of stressors experienced increased, participants’ resilience scores decreased [20]. While the correlation coefficient was relatively modest, this is not surprising given the subjectivity associated with stressful experiences, as well as the individual nature of psychological resilience [24, 25].

**Table 7:** Test-Retest Reliability Correlation Coefficients for PSSI and PSS-10

Total Sample	*r*_*s*_	95% CI	*n*
PSSI	0.78	(0.74, 0.82)	348
PSS-10	0.79	(0.74, 0.83)	349
Extreme Events Removed			
PSSI	0.78	(0.73, 0.82)	273
PSS-10	0.79	(0.74, 0.83)	278
Extreme Events Only			
PSSI	0.76	(0.64, 0.84)	71
PSS-10	0.67	(0.52, 0.78)	71

Note. Spearman’s rho (*r*_s_) correlation coefficients for non-parametric data reported

### Internal structure evidence

Results of an exploratory factor analysis (Table 8) supported our hypothesis that the PSSI would take the internal structure of an index, rather than a scale. That is to say that no clear groups of items emerged as viable subscales, making it appropriate to treat each item as an individual causal indicator of our underlying construct of post-secondary student stress.

**Table 8:** Factor Loadings for Items in PSSI

	*Component*										
*Item*	1	2	3	4	5	6	7	8	9	10	11
Academics											
1	.774										
2	.773										
3	.673										
4	.762										
5	.527									.450	
6	.490									.509	
7						.354				.501	
8	.450								.320		
9	.453								.490	.355	
10				.837							
11				.551							
Learning Environment											
12							.788				
13							.782				
14							.778				
15				.352							
16				.902							
17				.862							
Campus Culture											
18						.668					
19						.740					
20						.586					
21		.535				.514					
22		.582				.479					
23		.597				.327					
24											.807
25											.816
Interpersonal											
26			.767								
27			.782								
28		.324	.567								
29			.744								
30		.306	.501							.344	
31		.738	.319								
32		.584	.334								
33		.676	.327								
34		.699									
Personal											
35					.584						
36					.713						
37					.736						
38					.642						
39				.327				.340			
40					.388					.367	.336
41					.494					.332	
42								.894			
43		.309						.915			
44								.316	.481		
45									.747		
46									.428		
Eigenvalue	12.1	3.3	2.6	1.9	1.8	1.6	1.4	1.4	1.2	1.2	1.1

Note. KMO Statistic > 0.7 for all items

Barlett’s Test of Sphericity p < 0.001

Principal Components Analysis (Varimax rotation with Kaiser normalization)

With respect to test-retest reliability, a total of 365 students completed the second survey, and 350 responses were successfully matched using unique identifiers. Respondents completed the first iteration of the survey over the course of a four-week period. Invitations to complete the second survey were staggered in order to ensure that at least two weeks had passed between responses. As described above, we summed the absolute count of stressors experienced at each time point and examined the correlation between the average PSSI “scores” for the total sample. We sought the recommended minimum reliability coefficient of 0.7 [9]. The PSSI demonstrated strong test-retest reliability (*r*_s_ = 0.78; 95% CI 0.74, 0.82) comparable to that of the Perceived Stress Scale (PSS-10, *r*_s_ = 0.79; 95% CI 0.74, 0.83), which has been demonstrated to have consistent test-retest reliability averaging around 0.7 for a two-week interval [26].

While we hypothesized that students’ stress levels were likely to remain fairly static over a two-week period, we added a variable to the second survey in order to account for the possibility of a significantly stressful event producing a change in PSSI scores. Respondents were asked whether an event had occurred that caused them extreme stress since they submitted their last survey. Removing all respondents who indicated ‘yes’ (21.4%) from the dataset, we repeated our correlation analysis (*n* = 273), which revealed the test-retest reliability of the PSSI to be largely unchanged (*r*_s_ = 0.78; 95% CI 0.73, 0.82). Finally, we repeated the correlation analysis among only those who experienced an extremely stressful events (*n* = 71). Here, we saw a slight decrease in the correlation for the PSSI scores (*r*_s_ = 0.76; 95% CI 0.64, 0.84). Table 7 depicts all tests conducted for test-retest reliability.

## Discussion

By emphasizing mental health promotion and mental illness prevention, post-secondary institutions can provide students with the skills and resources needed to navigate stressors inherent to the post-secondary experience and thrive in the face of challenge. In order to develop and deliver efficacious promotion and prevention programming, however, there must first be a valid and reliable method of assessing post-secondary students’ exposure to stress. The Post-Secondary Student Stressors Index was created to fill this gap.

Unlike many of its predecessors, the PSSI was developed for students based on student input. We engaged four diverse samples of Canadian post-secondary students in the development, refinement, and testing of the instrument using a combination of qualitative and quantitative data collection approaches. Engaging students throughout the process facilitated the collection of robust content evidence for validity. Students were treated as subject matter experts, who were invested in helping us develop a holistic tool that accurately reflected student experiences of stress. In addition to content evidence, we collected response processes evidence by conducting one-on-one interviews with students to help us refine the tool and ensure that any item ambiguity and comprehension issues were addressed prior to moving forward to the testing stage.

Next, we conducted a pilot test to examine the internal structure of the tool as well as its relations to other variables. As expected, exploratory factor analysis revealed the PSSI to have the internal structure of an index, rather than a scale. That is to say that items on the PSSI did not “group” together, or load on distinct factors, as would be expected of a scale. The PSSI also demonstrated strong test-retest reliability over a two-week period, comparable to established measures like the PSS-10. Finally, the PSSI demonstrated good relationships with like measures of stress, distress, and resilience, in the hypothesized directions. As desired, the PSSI had a moderate correlation with the PSS-10, indicating that not only were we were successful in creating a tool that adequately measured stress, it also measured a specific *type* of stress (e.g., the stress experienced by students) rather than a more general assessment of stress. Similarly, the PSSI demonstrated a moderate correlation with the K10. Finally, the PSSI demonstrated a weak, negative correlation with the CD-RISC, indicating that students who experienced more stressors had a lower resilience score.

The PSSI is an inventory composed of 46 stressors across five major domains within the post-secondary setting: academics, the learning environment, campus culture, the interpersonal, and the personal. The tool evaluates each stressor by both severity and frequency. This dual-assessment approach will allow institutions to easily determine the priority areas (e.g., the most severe and frequently occurring stressors) for improvement on their campus, allowing for the unique targeting of mental health promotion and mental illness prevention programming.

### Limitations

Despite its strengths, there are some limitations to this study. Item pool development was conducted at a single, mid-sized Ontario university. During the item pool refinement stage, we intended to collect a sample of students from various regions in Canada in order to garner more regional representation in the development of the instrument. Despite our attempts to engage post-secondary students from across Canada using a snowball sampling approach for the consensus survey, we were only able to engage a handful of students from outside of Ontario. The pilot testing of the instrument was also conducted at one Ontario university. As a result, it is possible that this tool may be missing stressors that are prevalent in other regions of the country. Future research should continue to work towards the validation of this instrument among samples of students in different regions of the country and a more demographically varied sample in order to test and improve upon its generalizability.

## Conclusions

The PSSI was developed to fill the gap left by existing instruments that have unsuccessfully attempted to evaluate post-secondary student stress. Where previous instruments have been developed without taking students’ experiences into consideration, have demonstrated weak psychometrics, or have not collected sufficient evidence for validity, the PSSI excels. Overall, the PSSI demonstrated strong psychometric properties, suggesting that it is an effective tool for assessing post-secondary students’ exposure to stress. The ability to not only identify the sources of student stress (e.g., stressors), but also quantify the severity and frequency with which these stressors are experienced will provide institutions with a roadmap for the development of upstream approaches that can effectively target the sources of student stress and mental health problems on their campus.

## Data Availability

The datasets used for the current study are not publicly available due to required confidentiality and anonymity in compliance with the research ethics board.

## Appendix

“Appendix A – Factor Analysis” (Appendix A), appended to this article, details the results of the exploratory factor analysis conducted to evaluate the internal validity of the PSSI. The table depicts factor loadings, components, and other relevant statistics (i.e., eigenvalues, KMO statistic, Bartlett’s test).

## Abbreviations

CD-RISC: Connor-Davidson Resiliency Scale
CVI: Content Validity Index
K10: Kessler Psychological Distress Scale
NCHA II: National College Health Assessment II Survey
PSS-10: Perceived Stress Scale (10-item)
PSSI: Post-Secondary Student Stressors Index
